# The Effect of Lower-Body Blood Flow Restriction on Static and Perturbated Stable Stand in Young, Healthy Adults

**DOI:** 10.3389/fnhum.2021.756230

**Published:** 2021-10-22

**Authors:** Christina Willberg, Karen Zentgraf, Michael Behringer

**Affiliations:** ^1^Institute of Sport Sciences, Movement Sciences and Training in Sports, Goethe University Frankfurt, Frankfurt, Germany; ^2^Institute of Sport Sciences, Sports Medicine and Exercise Physiology, Goethe University Frankfurt, Frankfurt, Germany

**Keywords:** postural control, dynamic postural control, deoxygenation, muscular fatigue, BFR, perturbation

## Abstract

Muscular fatigue can affect postural control processes by impacting on the neuromuscular and somatosensory system. It is assumed that this leads to an increased risk of injury, especially in sports such as alpine skiing that expose the body to strong and rapidly changing external forces. In this context, posture constraints and contraction-related muscular pressure may lead to muscular deoxygenation. This study investigates whether these constraints and pressure affect static and dynamic postural control. To simulate impaired blood flow in sports within a laboratory task, oxygen saturation was manipulated locally by using an inflatable cuff to induce blood flow restriction (BFR). Twenty-three subjects were asked to stand on a perturbatable platform used to assess postural-related movements. Using a 2 × 2 within-subject design, each participant performed postural control tasks both with and without BFR. BFR resulted in lower oxygenation of the m. quadriceps femoris (*p* = 0.024) and was associated with a significantly lower time to exhaustion (TTE) compared to the non-restricted condition [*F*_(1,19)_ = 16.22, *p* < 0.001, η_*p*_^2^ = 0.46]. Perturbation resulted in a significantly increased TTE [*F*_(1,19)_ = 7.28, *p* = 0.014, η_*p*_^2^ = 0.277]. There were no significant effects on static and dynamic postural control within the saturation conditions. The present data indicate that BFR conditions leads to deoxygenation and a reduced TTE. Postural control and the ability to regain stability after perturbation were not affected within this investigation.

## Introduction

In many sport disciplines, athletes must respond quickly to changes in their environment and alter physical forces to maintain an optimal stance. In alpine skiing or speed skating, for example, there is a need to maintain an aerodynamic posture and to resist external disruptions of a stable upright postural state. The muscle activity thereby is mainly isometric and eccentric (Hofmann et al., 2001). Long-lasting, intensive isometric loading has been discussed as a potential cause of peripheral limitation of oxygen (O_2_) transport (Degens et al., 1998; Oranchuk et al., 2018). The resulting disparity between energy demand and consumption leads to muscular fatigue (Sjøgaard et al., 1988; Sutton, 1992; Goodall et al., 2014; Grassi et al., 2015), which is defined as a decrease in muscle contractility and, consequently, a loss of performance (Szmedra et al., 2001; Allen et al., 2008; Hettinga et al., 2016). Moreover, joint angles, intramuscular forces, and duration of contraction may also affect muscular blood flow (Konings et al., 2015). If joint angles of the hip or knee decrease or load duration and external forces increase, blood flow is attenuated, and muscle deoxygenation increases (Foster et al., 1999). This has been shown in skating (Foster et al., 1999) and alpine skiing in which a decrease in oxygen saturation of the quadriceps femoris could be observed to values below 10% (Behringer et al., 2018). Previous data also indicate that muscular fatigue impacts on the proprioceptive system by altering the processing of sensory information to the central nervous system, resulting in impaired motor control (Gribble and Hertel, 2004; Barandun et al., 2009; Enoka et al., 2011). Because maintenance of postural control (PC) depends on adequate functioning of the sensory and the motor system (Shumway-Cook and Woollacott, 2017), it can be assumed that PC relates to muscular deoxygenation. PC refers to the ability to maintain, achieve or restore the line of gravity within the base of support (Pollock et al., 2000, p. 405). Therefore, it involves the use of both predictive strategies (i.e., maintaining balance) and compensatory strategies (i.e., regaining balance) (Maki and McIlroy, 1997). With increasing fatigue, it becomes more difficult to maintain or regain an upright posture. The time duration until this position can no longer be maintained is defined as time to exhaustion (TTE), and is used to quantify fatigue in muscle endurance tasks (Pageaux et al., 2013; Van Cutsem et al., 2017).

Papa et al. (2015) have shown that muscular fatigue significantly affects stability and the risk of falls in older adults. This also holds for young and healthy individuals (Ghamkhar and Kahlaee, 2019). In sports, PC is seen as a prerequisite for enhancing performance (Garcia et al., 2011; Andreeva et al., 2020) and is therefore often included in performance diagnostics. Thereby, in static PC assessments, center of pressure displacements on a stable base of support are analyzed using posturography or force plates (Lin et al., 2008; Chaubet et al., 2012; Ahmadi et al., 2017). Even though these tools deliver insight into balance-related processes, they are limited when it comes to drawing conclusion on dynamic and perturbed movements. The importance of those movements in sports is evident: In alpine skiing, for example, athletes need to remain in a stable upright position while being disrupted continuously by the environment, equipment, or even their own movements. Perturbed-dynamic PC tests aim to simulate these perturbations and analyze the ability to regain stable erect posture after disruptions. Some are administered in anticipatory settings. However, because this reflects predictive/anticipatory rather than compensatory abilities, non-anticipatory settings have been used to investigate reactive postural corrections (Piscitelli et al., 2017). Nonetheless, these studies were performed mainly with older subjects or people with cognitive impairment (Sibley et al., 2015) and not in a sport-specific context.

The goal of the present study was therefore to investigate the ability to maintain and regain postural stability after external disturbance and to test whether this ability is changed by deoxygenation of the working muscles. It aimed to investigate whether increased deoxygenation and associated muscular fatigue would influence static and dynamic (perturbed) PC. Blood flow restriction (BFR), was used to decrease O_2_ saturation in the muscle. It was hypothesized that unperturbed and perturbed PC would deteriorate with increasing muscular deoxygenation.

## Materials and Methods

The study was approved in March 2018 by the local ethics committee of the Goethe University Frankfurt (Chair: Prof. Dr. Klein, 2018-34). All 23 subjects (13 female, 10 male; M_*age*_ = 26.2 female, 27.0 male) were informed about the procedure before the beginning of measurements and gave their written informed consent to participate. Three of them quit the project: two for personal reasons and one due to a lack of angle mobility hindering him in taking the required position. Because subjects were mainly sports students, the sample can be categorized as moderately to highly physically active according to WHO criteria (Pastuszak et al., 2014). None of the subjects was an elite athlete with practice time >6 h per day. All subjects were asked about their health status before the first appointment. Persons with hypotonic blood pressure, cardiovascular disease, or severe diseases that affect the quality of life were excluded. Those having had surgeries within the last 6 months, acute injuries, or the intake of perception-altering substances were also excluded. All subjects were advised not to change their activity-related habits during participation in this study. Body height and weight were measured on a scale (model 920, Seca GmbH & Co. KG, Hamburg, Germany) and subcutaneous fat *via* ultrasound (ACUSON X150, Siemens Medical Solutions United States, Mountain View, CA, United States). Table 1 reports anthropometric data.

**TABLE 1:** Descriptives of subjects after exclusion of three subjects.

	**Minimum**	**Maximum**	**Mean**	***SD***
Age (years)	23	34	26.55	2.56
Height (cm)	162	188	173.25	7.13
Weight (kg)	55	108	69.10	12.82
Thigh length (cm)	52	66	58.52	3.32
Subc. fat (mm)	0.6	5.4	2.21	1.0
Oc. pressure (mmHg)	115	225	148.25	22.96

*N = 20, Women = 11, Men = 9.*

Subc. fat = subcutaneous fat, *SD* = standard deviation, Oc. Pressure = occlusion pressure, mmHg = millimeters of mercury.

**FIGURE 1:**
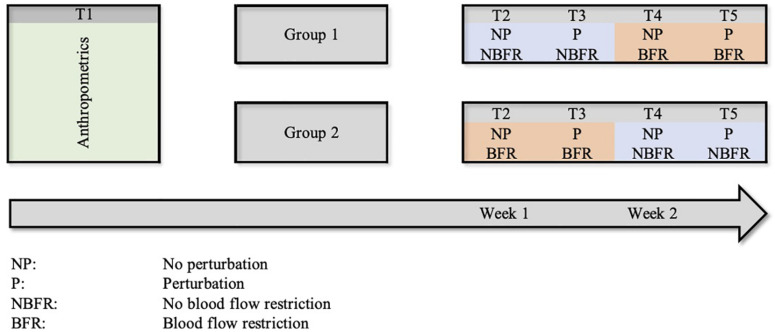
Within subject design of the study.

A 2 × 2 within subject design was used to investigate the effects of deoxygenation (with and without BFR) on static and dynamic postural control. Therefore, all 20 subjects participated in four sessions starting without perturbation (NP), followed by one measurement with perturbation (P) in both the BFR and NBFR conditions (Figure 1). To avoid sequence effects of the study design, subjects performed the measurements in two sequences of conditions distributed by block randomization. They were split into two equal sized groups, one starting with NBFR conditions, the other with BFR conditions. To eliminate carryover effects, washout periods of at least 48 h were used.

Before PC measurements, one session was used for anthropometric measurements and to define the subjects’ occlusion pressure. In each PC condition, subjects were asked to stand bipedally on a platform allowing free movements in mediolateral as well as anterior–posterior directions. They had to take a defined position that was controlled by a goniometer The time subjects could remain in this position was measured and used as TTE. PC was determined by the extent of translational platform movement. Additionally, PC in response to external perturbations was operationalized by the time to regain stable stand.

### Oxygen Saturation Monitoring and Occlusion Pressure

To assess O_2_ saturation, near-infrared spectroscopy (NIRS) measurement was used as a manipulation check. Due to restricted instrument availability, saturation was measured on a random sample basis in 50% of subjects. Therefore, the NIRS group consisted of 10 persons (7 female, 3 male) with a mean weight of 65.99 kg (*SD* = 8.01) and 170 cm (*SD* = 7.24) height. The device (MOXY-3, MOXY, Hutchinson, MN, United States) was placed on the quadriceps femoris. To define individual NIRS placement, the distance from the proximal patella margin to the anterior superior iliac spine was measured. Measurements of NIRS and subcutaneous fat were then taken at one third of the length between these anatomic landmarks. Following the protocol of Laurentino et al. (2018), occlusion pressure was measured in a lying position on the artery femoralis using 10 cm cuffs (UT 1317-L, ulrich GmbH & Co. KG, Ulm, Germany). All data were recorded on the leg that subjects preferred to shoot a ball with. Within BFR conditions, subjects were asked to stand in an upright position when cuffs were inflated. Afterward, they stood still for 60 s to guarantee muscle deoxygenation before the beginning of PC measurement.

### Measurement Setup

**FIGURE 2:**
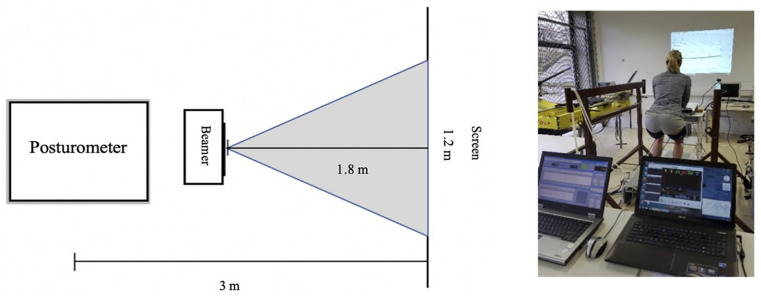
Test setup. Left: bird’s-eye view of the setup. Right: setup in the BFR condition.

To monitor whether subjects remained in the defined body posture, a goniometer (BN-GON-110-XDCR, Biopac Systems Inc, Goleta, CA, United States) was placed on their knee (vastus lateralis of quadriceps femoris, distal to caput fibulae). *Via* biofeedback, subjects could control their position during measurement, because knee angle was projected (LV-7275, Canon, Tokyo, Japan) on to a screen in front of them (see Figure 2). The knee joint angle was defined as 110° (±2.5°) flexion angle between thigh and shank measured at the dorsal side of the respective leg. This angle results in an intermediate high position that is seen to provide a stable position and is therefore often adopted by athletes in, for example, speed skating (Foster et al., 1999; Müller and Schwameder, 2003). Subjects were placed on a posturometer (Zeptoring Deutschland GmbH, Berlin, Germany) that recorded displacements in the anterior–posterior (antpost) and medial–lateral (medlat) direction with a 1000 Hz sampling rate. This made it possible to register the course of the center of pressure on the support surface. In the dynamic PC conditions, the plate of the posturometer was pushed from medial to lateral pneumatically (4.6 bar) to disturb the subject’s balance. The perturbations took place at 25, 50, 75, and 90% of the duration of the baseline measurement (NP) in the NBFR and BFR conditions of the corresponding participant. The timing of the perturbation was unknown for the subjects. All were told to maintain the position for as long as possible. Abortion criteria were either the loss of the target position three times within 10 s or a signal from the subjects that they could not hold the position any longer. Within the perturbation setting, same abortion criteria were used wherefore, in some cases (NBFR–75%: *n* = 2; 90%: *n* = 8; BFR – 90%: *n* = 5) subjects underwent less than 4 perturbations in their trial. If this was the case, only those perturbations which occurred before the measurement was stopped were considered for analysis. The duration from start to measurement abortion was measured and defined as TTE.

### Data Processing

Data processing was carried out using MATLAB (R2018b, The MathWorks, Inc., Natick, MA, United States). As part of the data preprocessing, the raw data of the NIRS and the posturometer were first synchronized. Due to the different recording frequencies of the measuring instruments (MOXY 2 Hz, posturometer 1000 Hz), the NIRS data were subsequently linearly interpolated to 1000 Hz. Both, the antpost and the medlat data generated by the posturometer, were used to calculate the subjects’ postural sway as the time-average of the instantaneous change in displacements in two directions. Therefore, the distance between two successive data points (antpost_*T* = i_, antpost_*t* = *i* + 1_) in antpost and medlat direction was calculated and added over the entire course of the data. Since the measurement duration varied, data had to be relativized over the test duration (n) using the formula below.


$$r\,e\,l\,a\,t\,i\,v\,e\,p\,o\,s\,t\,u\,r\,a\,l\,s\,w\,a\,y=\frac{\sum_{t=0}^{t=n}\sqrt{\begin{array}{cccc} {\left(a\,n\,t\,p\,o\,s\,t_{t=i+1}-a\,n\,t\,p\,o\,s\,t_{t=i}\right)}^{2}+ & & & \\ {\left(m\,e\,d\,l\,a\,t_{t=i+1}-m\,e\,d\,l\,a\,t_{t=i}\right)}^{2} & & & \end{array}}}{n}$$

The restoration of postural control (referred to in the rest of the manuscript as re-stabilization) was defined as the time subjects needed from the start of perturbation until getting back to a “stable,” bipedal stand. “Stable” stand was determined based on the subjects’ time-average of postural sway in phase 2 of the NBFR NP condition because the plate displacement was the lowest in this phase. We determined the time, until the subjects get back to “stable” stand (phase 2 time-averaged postural sway ± 2^∗^SD) as re-stabilization time.

Because one could assume that disturbance of the stable stand would have a short-term influence on O_2_ saturation due to a shortly altered position, O_2_ saturation was also calculated at the time of perturbation and shortly after (mean O_2_ within 3 s after perturbation).

Near-infrared spectroscopy measurements under load are associated with a large dispersion of the measured values. To minimize the influence of any erroneous measurements, a moving average was calculated over 3,000 data points (3 s), whereby the mean value of an phase of 3,000 data points centered at the current time point was calculated continuously.

### Statistics

SPSS (IBM SPSS Statistics, Version 24, Chicago, IL, United States) was used for the statistical analysis. Results were displayed using Jamovi (Jamovi project, Version 0.9, 2018) to illustrate mean values and 90% confidence intervals. Data were tested for normal distribution using the Shapiro–Wilk test and for violation of sphericity using Mauchly’s test (*p* > 0.05). In line with the test design (within-subject) and hypotheses, variance analyses (multifactorial repeated-measures ANOVAs) were calculated to detect mean value differences between the conditions. First, relative postural sway was analyzed in general, using a 2 × 2 ANOVA with PC and O_2_ saturation conditions being the factors. To further analyze relative postural sway during the course of the measurement a 2 × 5 ANOVA was used (PC conditions, measurement intervals). The segmentation into five intervals was made under consideration of the perturbation times and thus by the duration of the respective baseline conditions. Phase 1 comprised 0–20% of the duration of the NP setting; Phase 2, 20–40%; Phase 3, 40–60%; Phase 4, 60–80%; and Phase 5, 80–100%. This ensured that one perturbation was mapped in each of Phases 2–5. In a second step, re-stabilization time and O_2_ saturation at the points of perturbation were analyzed using a 2 × 4 ANOVA (O_2_ saturation conditions, points of perturbation). Also, oxygenation status before and directly after the point of perturbation was analyzed using a 2 × 2 × 4 ANOVA with O_2_ saturation conditions, time point of NIRS measurement (pre, post perturbation) and points of perturbations. To analyze mean differences of TTE, a 2 × 2 ANOVA with PC and O_2_ saturation conditions was conducted. Finally, as manipulation check, O_2_ saturation was analyzed in PC and O_2_ saturation conditions using a 2 × 2 ANOVA. As NIRS measurement was only conducted in *n* = 10 subjects, only those values are included into analysis where O_2_ saturation was the outcome variable. Effect sizes of variance analyses are expressed as η_*p*_^2^. In case of significant interaction or main effects, *post hoc* testing was carried out with *t* tests using Bonferroni correction of the alpha level. Jamovi was used to create graphs and evaluate the *post hoc* tests in variance analyses with more than two factors. The significance level was set at *p* < 0.05 across all calculations. Results are all given in absolute numbers. Concerning O_2_ saturation, the “%” marks only the unit of O_2_ saturation, because this is usually displayed as a percentage. To avoid misinterpreting non-significant results, the absence of an effect was controlled with equivalence tests (Lakens, 2017). Paired-samples *t* tests were calculated as paired two one-sided tests (TOST-P) for the analysis of relative postural sway (Mara and Cribbie, 2012). The smallest effect size of interest was set at 0.46, which was the effect size of mediolateral sway reported by Barbieri et al. (2019) when investigating the effects of ankle muscle fatigue on postural sway.

## Results

**FIGURE 3:**
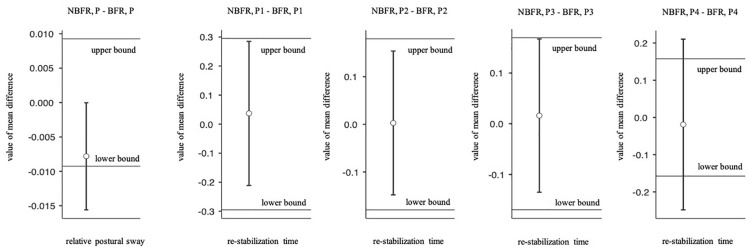
Paired two one-sided *t* tests for equivalence (TOST-P). Boundaries and 90% CI of the relative postural sway and re-stabilization time. NBFR = No BFR, NP = No perturbation, P = Perturbation.

Mauchly’s test of sphericity indicated that the assumption of sphericity is met. There is no significant interaction between PC and O_2_ saturation conditions [*F*_(1,19)_ = 3.8, *p* = 0.07, η_*p*_^2^ = 0.17]. Relative postural sway did not differ significantly between NBFR and BFR conditions [*F*_(1,19)_ = 0.92, *p* = 0.35, η_*p*_^2^ = 0.05]. However, the result is not equivalent to zero when calculating TOST-P with the defined effect size of interest [upper bound: *t*(19) = –3.79, *p* < 0.01, lower bound: *t*(19) = 2.32, *p* = 0.38; Figure 3]. As expected, relative postural sway was significantly lower in NP than in P conditions [*F*_(1,19)_ = 41.1, *p* < 0.01, η_*p*_^2^ = 0.68].

**FIGURE 4:**
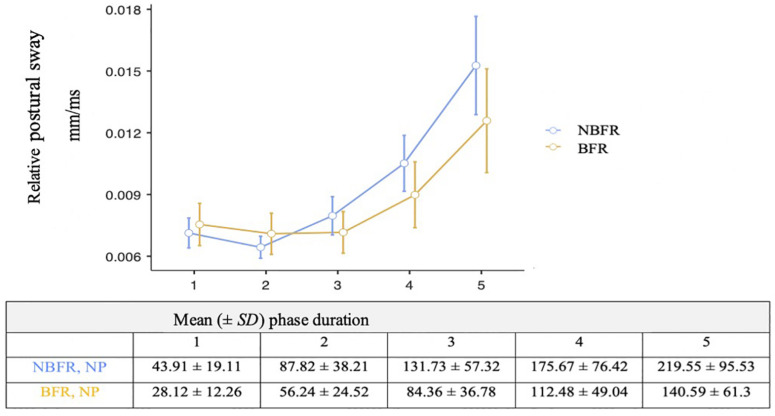
Analysis of relative postural sway and measurement duration in the non-perturbated condition. Mean value comparison and 90% CI of the relative postural sway. NBFR = No BFR. *N* = 20 in all phases.

Concerning time effects in stable bipedal stand, the Greenhouse–Geisser adjustment was used to correct for violations of sphericity. There is a significant interaction [*F*_(4,76)_ = 6.11, *p* = 0.06, η_*p*_^2^ = 0.24] concerning PC conditions and measurement duration (Figure 4). While relative postural sway is lower in NBFR condition in the first intervals, differences in O_2_ saturation increase during the course of the measurement with NBFR achieving higher values than BFR (*p*_*bonf*_ = 0.01, dif_*mean*_ = –0.01). There is a main effect of time, suggesting that the measurement duration influences relative postural sway values [*F*_(4,76)_ = 33.19, *p* < 0.01, η_*p*_^2^ = 0.02].

**FIGURE 5:**
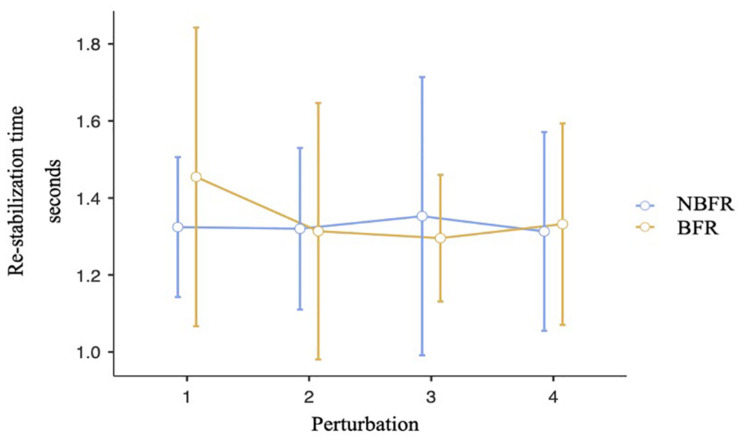
Analysis of the re-stabilization time at the points of perturbation. Mean value comparison and 90% CI. NBFR = No BFR.

Analyzing the re-stabilization time, Mauchly’s test of sphericity indicated that the assumption of sphericity is met. There is no interaction effect between the time of perturbation and the O_2_ saturation conditions [*F*_(3,21)_ = 0.52, *p* = 0.67, η_*p*_^2^ = 0.07, Figure 5]. Also, there are no significant differences between NBFR and BFR conditions [*F*_(1,7)_ = 0.04, *p* = 0.84, η_*p*_^2^ = 0.01] and timepoints of perturbation [*F*_(3,21)_ = 0.29, *p* = 0.83, η_*p*_^2^ = 0.04]. Calculating TOST-P, the effects of perturbation one to three are equivalent to zero [p1: upper bound: *t*(19) = –1.8, *p* = 0.04, lower bound: *t*(19) = 2.32, *p* = 0.02, p2: upper bound: *t*(19) = –2.03, *p* = 0.03, lower bound: *t*(19) = 2.09, *p* = 0.03, p3: upper bound: *t*(17) = –1.77, *p* = 0.05, lower bound: *t*(17) = 2.12, *p* = 0.02]. Regarding perturbation four, results of TOST-P do not show equivalence [upper bound: *t*(7) = –1.46, *p* = 0.09, lower bound: *t*(7) = 1.14, *p* = 0.15]. The assumption that the effect is equivalent to zero can therefore not be proven for the last interval. Although the assumption of postural changes leading to short-time re-oxygenation cannot be confirmed [*F*_(1,6)_ = 2.1, *p* = 0.2, η_*p*_^2^ = 0.26], significant differences between the points of interruption were detected [*F*_(3,18)_ = 5.02, *p* = 0.01, η_*p*_^2^ = 0.46]. During the first perturbation O_2_ saturation values are higher than during the last one (*p*_*bonf*_ = 0.01, dif_*mean*_ = 9.33%).

**FIGURE 6:**
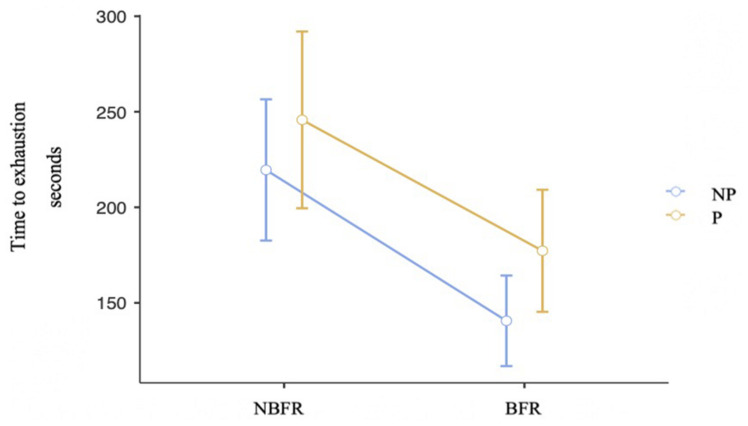
Analysis of the TTE in PC and O_2_ saturation conditions. Mean value comparison and 90% CI of the time to exhaustion in all conditions. NP = No perturbation, P = Perturbation, NBFR = No BFR.

Because O_2_ saturation is assumed to be an important factor influencing muscular fatigue, TTE was analyzed. There is no violation of sphericity. As displayed in Figure 6, there are no interaction effects between PC and O_2_ saturation conditions [*F*_(1,19)_ = 0.24, *p* = 0.63, η_*p*_^2^ = 0.01]. TTE is shorter in BFR than in NBFR conditions [*F*_(1,19)_ = 16.22, *p* < 0.001, η_*p*_^2^ = 0.46, dif_*mean*_ = 73.7 s]. Regarding PC conditions, subjects can maintain the position longer (dif_*mean*_ = 31.4 s) in the conditions where perturbation occur [*F*_(1,19)_ = 7.28, *p* = 0.01, η_*p*_^2^ = 0.27, dif_*mean*_ = of –31.4 s].

As a manipulation check, the saturation status of 10 subjects was analyzed. Mauchley’s test of sphericity indicated that the assumption of sphericity is met. There is no significant interaction effect between PC and O_2_ saturation conditions [*F*_(1,9)_ = 2.95, *p* = 0.12, η_*p*_^2^ = 0.25]. As expected, O_2_ saturation is significantly higher in NBFR conditions (dif_*mean*_ = 17.5%) than in BFR conditions [*F*_(1,9)_ = 7.4, *p* = 0.02, η_*p*_^2^ = 0.45]. PC conditions do not differ [*F*_(1,9)_ = 0.52, *p* = 0.49, η_*p*_^2^ = 0.06; Supplementary Figure 1].

## Discussion

This study aimed to investigate the ability to maintain and regain stability after external disturbance and to test whether this ability is changed by deoxygenation and the associated fatigue of the working muscle. As expected, the external pressure on the tissue caused by the application of pneumatic cuffs led to reduced O_2_ saturation and premature fatigue defined as a reduced TTE. However, changes in the subjects’ sway due to decreased O_2_ saturation were not significant in static and perturbed-dynamic measurements.

A decreased TTE in the BFR conditions may be explained by the processes of muscular fatigue. Due to the lack of O_2,_ there is a failure in oxidative capacity, leading to a fast decline of force in type-I muscle fibers (Scott et al., 2014). The recruitment of fast-twitch fibers to maintain muscle function leads to an accelerated accumulation of metabolites (Loenneke et al., 2011), because the organism has to resort to other energy-producing metabolic processes to maintain the adenosine triphosphate (ATP) supply. Instead of mitochondrial ATP synthesis, anaerobic glycolysis becomes the main energy source. Due to this shift, phosphate accumulates that impairs the muscular work (for an overview, see Allen et al., 2008). Accordingly, the assumption that deoxygenation would lead to a decreased TTE could be confirmed in this study. Interestingly, TTE was significantly lower in the static condition than in the perturbed-dynamic condition.

There are different possible explanations for this observation: First, changes in position during the perturbation could have influenced O_2_ saturation. Due to compensatory body movements, joint angles may have increased for a short time and thereby decreased contraction-induced pressure on the arterioles. Even though the main effect regarding O_2_ saturation in PC conditions was not significant, differences between NBFR-NP and NBFR-P conditions could be seen, which supports this hypothesis. Second, it could be explained by the agonistic and antagonistic muscle activation. If the m. quadriceps femoris is the agonist for the isometric task, the antagonists (i.e., hamstrings) primarily need to work eccentrically to inhibit translation from medial to lateral (Blackburn et al., 2000). Within this mechanism, the quadriceps femoris does not have to perform extra work when a perturbation occurs. Instead, there might be a short-term force release, resulting in a relaxation of the quadriceps femoris. In both explanations, the relaxation of the quadriceps would lead to a short-time blood and O_2_ supply that might help to resist muscular fatigue. To investigate these assumptions, differences in O_2_ saturation shortly before and after the perturbations were calculated. No changes concerning the NIRS measurement could be detected. This might be explained by the insufficient recording sensitivity and sampling frequency of the NIRS device used in this investigation. Additionally, it should be noted that only values from 10 subjects were included in the calculation, therefore the results should be regarded as an indication, not as proof. However, throughout the perturbations a deoxygenation could be seen, especially in the last phases. Whereas there were only small differences in O_2_ saturation in the NBFR trials, the BFR conditions showed a trend toward decreasing saturation over time. This supports the hypothesis that re-oxygenation due to changed positions in the NBFR settings might have occurred. The fact that the perturbated NBFR conditions showed the highest O_2_ saturation also argues in favor of this explanation. To obtain more robust answers regarding the underlying mechanisms, future investigations should enhance the control of the joint position. One option might be to include data from the goniometer. Additionally, the use of a NIRS device that allows for a higher sampling frequency is recommended.

A third explanation for the increasing TTE within the perturbation setting might be the mental distraction of the subjects due to the perturbations. The additional re-stabilization task might have led to an increase in motivation in those conditions. Additionally, a shift of focus might have occurred when subjects were trying not only to remain in one position but also to react quickly and regain stability after perturbation. Even though this was not controlled within this investigation, psychological or motivational effects could also be an explanation for the varying TTE between conditions.

As mentioned in the introduction, muscular fatigue is supposed to have a negative effect on PC due to the impairment of proprioception and kinesthesia. Previous data indicate that fatigue alters the sensitivity of the muscle spindles, leads to a deterioration of afferent feedback, and reduces contraction force (Satas et al., 2020), resulting in a measurable decline of PC (Papa et al., 2015). In the present study, there was no significant change in relative postural sway between O_2_ saturation conditions in the NP task. However, the results of the equivalence test show that the effect cannot be declared irrelevant. Reasons for these contradictory statements are that either the power of the study was too low to show existing effects with a 5% probability of error or the limits of the equivalence test were underestimated. Although the effect of fatigue on PC has already been investigated many times, effect sizes have rarely been reported, which makes it difficult to define boundaries.

Within each condition, relative postural sway increased during the course of the measurement. It should be noted here that the shortened TTE (e.g., in the BFR condition) must be taken into account when comparing the plate displacement over time. Interestingly, the relative postural sway was higher in the NBFR condition than in the BFR conditions. This phenomenon could be explained by different fatiguing processes. In the NBFR conditions an increased muscle tremor could be observed when muscular fatigue increased, which might be a reaction of the neuromuscular system to muscular work (Proske, 2019). However, the subjects were able to maintain the position despite the tremor for a long time in the NBFR. Although subjects reported that BFR was at first seen as less exhausting, the measurement was stopped in most of the cases before a tremor occurred. In this conditions fatigue and the failure to maintain posture started more suddenly and were sometimes accompanied by pain. This raises the question as to how far BFR influences somatosensory and pain perception due to the mechanical stimulation of, e.g., nerve bundles of effector afferences. Because peripheral nerves are considered to be particularly pressure-sensitive, further investigations could focus on the extent of the impairment by attaching BFR cuffs. It is conceivable that exceeding a somatosensory sensitivity threshold, which could result in an exacerbation of pain or fatigue perception, might be one explanation for an earlier and more sudden termination. As discussed before, the shift in focus due to cuff application and the resulting change in sensory feedback could be another reason for the late, but more rapid onset of fatigue. Because subjects were not accustomed to the application of BFR cuffs, their focus might have been more on the cuffs rather than on the biofeedback task. A longer familiarization within the test settings could help to control this parameter.

To analyze time effects, total times were subdivided into phases. However, because the measurement duration was lower in BFR conditions than in the corresponding NBFR conditions and subjects had more problems keeping their balance with increasing time, the comparability of the phases and the significance of the results is limited. Under BFR conditions, increased fatigue and the deterioration of PC are to be expected. In the context of this study, it is therefore comparable only directly up to the point at which the corresponding BFR conditions were aborted by the test subjects. However, in a situation without perturbations, deoxygenation does not seem to influence relative postural sway.

Because inferences from static to dynamic PC are of limited validity (Morrison and Sosnoff, 2010; Sibley et al., 2015), this study additionally investigated the response to external perturbation. A decline in the ability to restabilize was hypothesized in the BFR setting. However, significant differences between the O_2_ saturation conditions weren’t found. Additionally, exhaustion (operationalized by enhanced translatory plate movement) was not a factor influencing re-stabilization time. For the first three perturbations, this result could be confirmed by equivalence testing. Concerning the fourth perturbation, the equivalence test was non-significant. However, as some subjects could not remain the position until the last perturbation, the sample size of TOST-P is smaller concerning the last perturbation. There were only eight persons, who resisted all perturbations in both conditions. It is therefore important to emphasize that although the results can indicate tendencies, these should be examined further in order to be able to make distinctive statements. One reason for the absence of a decline in re-stabilization ability in this study might be the different activation patterns of the musculature within the static stand and the re-stabilization task. As described above, the disruption of PC changes the joint angles of the limbs. Therefore, muscle activation patterns are altering in the trunk and lower extremities, resulting in increased coactivation of muscles in these areas (Horak, 2006). Also, more motor units are recruited, and the innervation rate increases (Pollock et al., 2000). Whereas the quadriceps femoris primarily provides isometric strength in the stance phases, more muscle groups might be activated as a result of disruption, and this would relieve the quadriceps femoris. The muscular work applied to maintain a stable position against disruptions is primarily eccentric (Hofmann et al., 2001). In comparison to a stable position, PC disruption thus makes different requirements on the musculature. This might explain the non-significant change of re-stabilization time in fatiguing tasks. Moreover, it is conceivable that no additional O_2_ consumption results from the perturbation, because the stabilizing muscles contract only briefly in the perturbation situation (average re-stabilization time < 1.4 s) and thus rely primarily on anaerobic metabolism for energy production—which acts independently from the O_2_ supply. Thus, there would be no additional O_2_ consumption due to the re-stabilization task. Another explanation could be the lack of muscular fatigue of the stabilizing musculature in the design of the study. Whereas an athlete’s deep musculature has to work permanently for stabilization during a downhill run in alpine skiing, these muscles were activated less in this test setup because there were only four perturbations. The state of fatigue of the stabilizing musculature can therefore be considered as relatively low in this study. To intensify the impact, shortening the time phases between the perturbations could be one option, whereby it would have to be ensured that the test persons reach a stable state before further perturbation is triggered.

This work does reveal some limitations regarding the measurement setup and the data processing. Concerning the predefined position, it was notable that the hip angle varied depending on the flexibility of the ankle joint, thereby resulting in different hip angles. Thus, by using biofeedback, it was possible to standardize the knee angle but not the body position across subjects and settings. This might have influenced the manipulation of the subjects’ blood flow. A further problem was the low acquisition rate of the NIRS that resulted in high variances. Although the reliability of the measurement technique seems to be given under such conditions, how far external factors such as muscle contractions influence the device is unclear. It would also be useful to determine the latency of the NIRS device and to include appropriate correction factors in the corresponding data sections (e.g., to investigate the effects of PC disruption more closely). Again, it should be noted, that within this study NIRS measurement was conducted on ten participants. An interpretation of the NIRS data should therefore be made cautiously. However, this study confirmed that BFR is a valid method to investigate the effects of muscle deoxygenation. Relative postural sway is not impaired by reduced O_2_ saturation in the working muscle neither in static nor in perturbed-dynamic conditions.

## Conclusion

Muscular deoxygenation during prolonged isometric muscle activity and the associated muscular fatigue influenced TTE, whereas static and dynamic postural control were not impaired directly by cuff application. In the dynamic PC conditions, TTE was not affected by reduced O_2_ saturation in the BFR settings. Furthermore, due to the perturbation of the stable stand, TTE could be delayed in the BFR and NBFR test settings. This may be explained by a short-time load change as well as by a possible relief and reoxygenation through the change of body position. Regarding the PC, manipulation of O_2_ saturation did not differ significantly between the static and dynamic settings. An increase in postural sway was observed during ongoing muscular fatigue in all conditions. If static PC is considered, this investigation confirms the assumption that stability deteriorates with decreased O_2_ saturation, which is associated with muscular fatigue. This effect is more pronounced in BFR conditions than in NBFR conditions. Considering the re-stabilization task, even when the duration of the stable stand increased and muscular fatigue was greater, no change in the re-stabilization time could be shown. This leads to the assumption that muscular deoxygenation does not affect performance within a non-anticipated re-stabilization task.

## Data Availability Statement

The raw data supporting the conclusions of this article will be made available by the authors, without undue reservation.

## Ethics Statement

The studies involving human participants were reviewed and approved by Local Ethics Committee of the Goethe University Frankfurt (Chair: Klein, Grant Number: 2018-34). The patients/participants provided their written informed consent to participate in this study. Written informed consent was obtained from the individual(s) for the publication of any potentially identifiable images or data included in this article.

## Author Contributions

All authors prepared the set up together. CW carried out the measurements and data analyses, and also wrote the manuscript. MB and KZ helped interpreting the data and checked the manuscript drafts several times.

## Conflict of Interest

The authors declare that the research was conducted in the absence of any commercial or financial relationships that could be construed as a potential conflict of interest.

## Publisher’s Note

All claims expressed in this article are solely those of the authors and do not necessarily represent those of their affiliated organizations, or those of the publisher, the editors and the reviewers. Any product that may be evaluated in this article, or claim that may be made by its manufacturer, is not guaranteed or endorsed by the publisher.

## Supplementary Material

Supplementary Figure 1 Analysis of the O_2_ saturation in PC and O_2_ saturation conditions. Mean value comparison and 90% CI. NP = No perturbation, P = Perturbation, NBFR = No BFR.
