# Supplementary figures and images for: The Effect of Lower-Body Blood Flow Restriction on Static and Perturbated Stable Stand in Young, Healthy Adults
Source: Front Hum Neurosci. 2021 Oct 22;15:756230. doi: 10.3389/fnhum.2021.756230 (PMC8570169; doi:10.3389/fnhum.2021.756230)

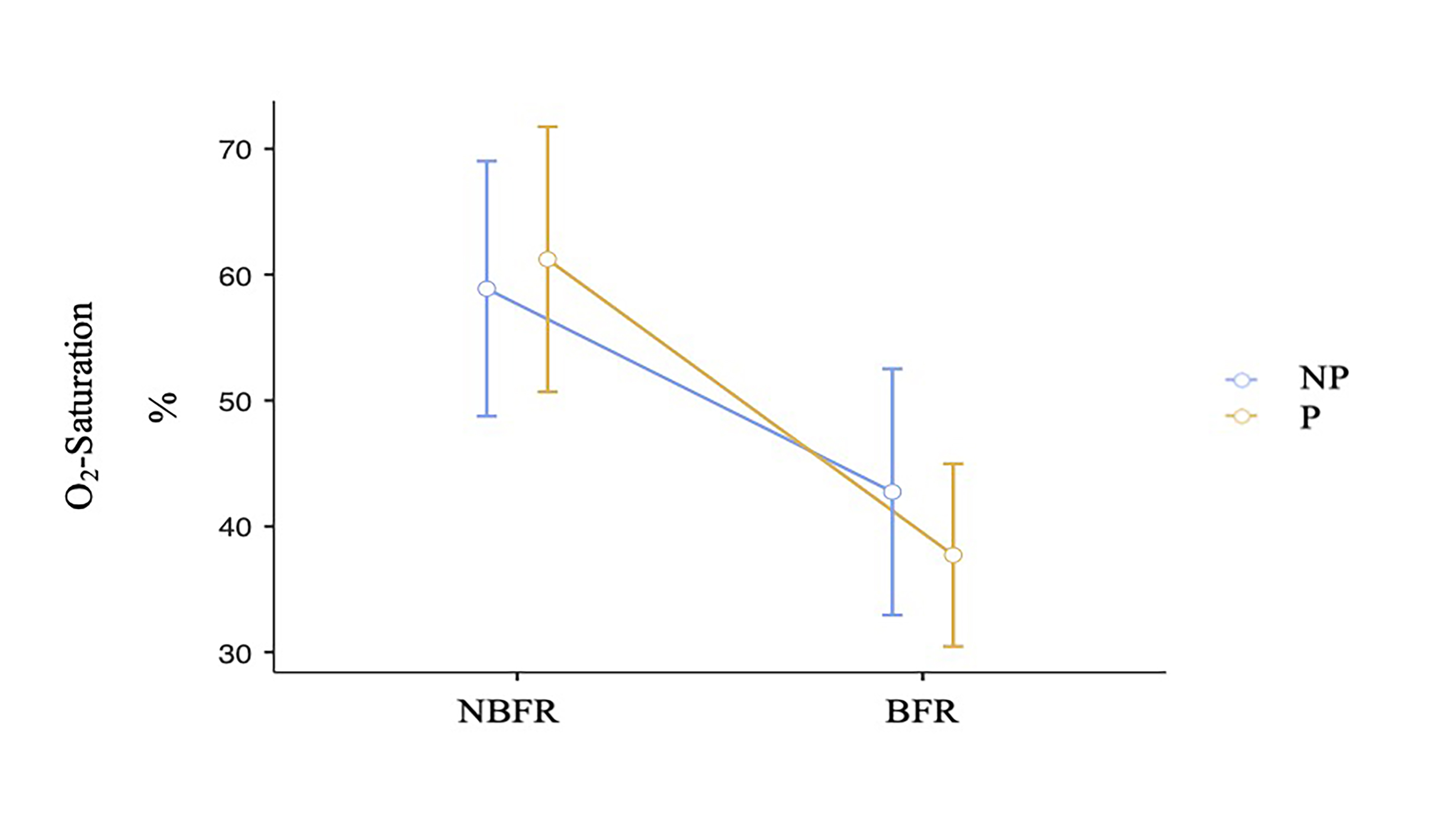

Supplement: Supplementary Figure 1 — Analysis of the O2 saturation in PC and O2 saturation conditions. Mean value comparison and 90% CI. NP = No perturbation, P = Perturbation, NBFR = No BFR. [file Image_1.JPEG]
